# Supplementary material for: Transcriptomic Characterization of Genes Regulating the Stemness in Porcine Atrial Cardiomyocytes during Primary In Vitro Culture
Source: Genes (Basel). 2023 Jun 4;14(6):1223. doi: 10.3390/genes14061223 (PMC10297922; doi:10.3390/genes14061223)
Supplement: Supplementary file 1 [file genes-14-01223-s001.zip › genes-2420763-supplementary.pdf]

Table S1. Symbols, changes in expression and adjusted p-values of 10 most deregulated genes in 7th, 15th and 30th day of cell culture derived from the fragment of right arial wall.

**Right atrial wall D7/D0**			
**Gene symbol**	**Gene name**	**Ratio**	**Adj. p-value**
SFRP2	secreted frizzled-related protein 2	91.25293	3.41E-06
PRRX1	paired related homeobox 1	73.52752	2.28E-06
SNAI2	snail family transcriptional repressor 2	10.69955	1.71E-05
RACGAP1	Rac GTPase activating protein 1	8.056284	0.000174048
RUNX1	RUNX family transcription factor 1	7.688494	0.004060851
TET1	tet methylcytosine dioxygenase 1	7.264942	4.04E-05
PAX6	paired box 6	6.925844	1.44E-05
HIF1A	hypoxia inducible factor 1 subunit alpha	5.643026437	0.002096652
DOCK7	dedicator of cytokinesis 7	5.601316092	2.50E-05
KLF10	Kruppel-like factor 10	5.483210429	0.002867812
**Right atrial wall D15/D0**			
**Gene symbol**	**Gene name**	**Ratio**	**Adj. p-value**
PRRX1	paired related homeobox 1	58.0526	2.66E-06
SFRP2	secreted frizzled-related protein 2	45.91556	6.50E-06
TET1	tet methylcytosine dioxygenase 1	8.274278	3.21E-05
RACGAP1	Rac GTPase activating protein 1	7.824558	0.000196515
SNAI2	snail family transcriptional repressor 2	6.952879	3.57E-05
DOCK7	dedicator of cytokinesis 7	6.259523594	2.05E-05
KLF10	Kruppel-like factor 10	6.154489147	0.002262327

HIF1A	hypoxia inducible factor 1 subunit alpha	5.911749057	0.001975997
KITLG	KIT ligand	5.75209235	3.76E-05
ID4	inhibitor of DNA binding 4, HLH protein	5.201814564	0.000160577
**Right atrial wall D30/D0**			
**Gene symbol**	**Gene name**	**Ratio**	**Adj. p-value**
PRRX1	paired related homeobox 1	55.30691	2.48E-06
SFRP2	secreted frizzled-related protein 2	32.01501	9.50E-06
TBX3	T-box transcription factor 3	12.71104	1.77E-05
FGF2	fibroblast growth factor 2	9.279203494	7.86E-05
TET1	tet methylcytosine dioxygenase 1	8.381566	3.28E-05
HIF1A	hypoxia inducible factor 1 subunit alpha	7.269599434	0.001293025
DOCK7	dedicator of cytokinesis 7	7.108950161	1.68E-05
KITLG	KIT ligand	6.528684668	3.10E-05
RUNX1	RUNX family transcription factor 1	5.432937	0.01060866
LIF	LIF interleukin 6 family cytokine	5.186656838	0.000310085

Supplementary Table S2. Symbols, changes in expression and adjusted p-values of 10 most deregulated genes in 7th, 15th and 30th day of cell culture derived from the fragment of right arial appendage.

**Right atrial appendage D7/D0**			
**Gene symbol**	**Gene name**	**Ratio**	**Adj. p-value**
SFRP2	secreted frizzled-related protein 2	61.19893155	1.81E-07
PRRX1	paired related homeobox 1	24.27644802	9.54E-07
RACGAP1	Rac GTPase activating protein 1	15.49744849	6.78E-06
FANCD2	FA complementation group D2	9.391782881	3.69E-06

SNAI2	snail family transcriptional repressor 2	8.575694836	1.52E-05
ASPM	assembly factor for spindle microtubules	6.994969446	4.82E-06
HIF1A	hypoxia inducible factor 1 subunit alpha	6.557233528	1.92E-05
KLF10	Kruppel-like factor 10	6.553449891	2.20E-06
DOCK7	dedicator of cytokinesis 7	5.783443744	2.49E-06
PAX6	paired box 6	5.268159343	1.35E-05
**Right atrial appendage D15/D0**			
**Gene symbol**	**Gene name**	**Ratio**	**Adj. p-value**
SFRP2	secreted frizzled-related protein 2	114.5021533	1.15E-07
PRRX1	paired related homeobox 1	33.31632816	6.94E-07
HIF1A	hypoxia inducible factor 1 subunit alpha	7.752175853	1.58E-05
KLF10	Kruppel-like factor 10	7.474406056	1.85E-06
DOCK7	dedicator of cytokinesis 7	6.587462279	2.15E-06
RACGAP1	Rac GTPase activating protein 1	4.803151211	0.000137476
TET1	tet methylcytosine dioxygenase 1	4.529717852	8.53E-05
KIT	KIT proto-oncogene, receptor tyrosine kinase	4.091056982	2.47E-05
MED14	mediator complex subunit 14	3.985152941	0.000106386
TBX3	T-box transcription factor 3	3.714752736	1.51E-05
**Right atrial appendage D30/D0**			
**Gene symbol**	**Gene name**	**Ratio**	**Adj. p-value**
SFRP2	secreted frizzled-related protein 2	110.9425022	1.11E-07
PRRX1	paired related homeobox 1	34.20995421	5.75E-07

HIF1A	hypoxia inducible factor 1 subunit alpha	6.440413457	2.52E-05
DOCK7	dedicator of cytokinesis 7	5.876689307	2.54E-06
KLF10	Kruppel-like factor 10	5.572430881	3.39E-06
TBX3	T-box transcription factor 3	5.538340048	4.18E-06
PAX6	paired box 6	4.958727004	1.99E-05
TET1	tet methylcytosine dioxygenase 1	4.764415595	7.24E-05
WNT2B	Wnt family member 2B	4.417734575	0.00014699
NF1	neurofibromin 1	3.729415734	8.77E-06
